# Perspectives on midwife-led care as a solution to reduce obstetric violence in health facilities in Ghana

**DOI:** 10.3389/fgwh.2025.1654504

**Published:** 2026-01-27

**Authors:** Gloria Senkyire, Ephraim Senkyire, Ernestina Asiedua, Emmanuel Lamptey, Victor Tawose-Adebayo, Rullmann Twi Owusu

**Affiliations:** 1 Department of Accountancy, Faculty of Business and Management Studies, Sunyani Technical University, Sunyani, Ghana; 2 Department of Nursing and Midwifery, Ga West Municipal Hospital-Ghana Health Service, Amasaman-Accra, Ghana; 3 Department of Maternal and Child Health, School of Nursing and Midwifery, University of Ghana, Legon-Accra, Ghana; 4 Department of Biomedical Sciences, School of Basic and Biomedical Sciences, University of Health and Allied Sciences, Ho, Ghana; 5 Behavioural Training, Conceptcare Disability, Sydney, NSW, Australia; 6 Department of Marketing and Supply Chain Management, School of Business, University of Cape Coast, Cape Coast, Ghana

**Keywords:** maternal healthcare, maternal mortality, maternity care, midwifery-led care, obstetric violence

## Abstract

**Background:**

Ghana's maternal mortality rate is substantially higher, well above the global target of 70 per 100,000 births. Despite high antenatal care attendance, less than seventy per cent of births are attended by skilled personnel, with some women opting for home births with unskilled attendants due to obstetric violence. Obstetric violence and the abuse inflicted by healthcare workers on pregnant women during childbirth deter women from facility-based births and exacerbate maternal mortality.

**Objective:**

To explore how implementing midwife-led care can mitigate obstetric violence and enhance maternal health outcomes in Ghana through a literature-informed perspective.

**Approach:**

Existing evidence was drawn from primary and secondary sources, including the World Health Organisation and the International Confederation of Midwives. Literature was synthesised to identify common patterns across studies.

**Results:**

Implementing midwife-led care, which emphasises a bio-psycho-social approach and supports women's autonomy and comfort, can mitigate obstetric violence and enhance maternal health outcomes. Scaling up midwife-led primary care and providing training in humanised care at health facility levels are essential steps toward this goal.

**Conclusion:**

Midwife-led care is a valid and evidence-based approach, proven effective in multiple countries. Implementation is feasible in Ghana but requires system readiness and stakeholder engagement.

## 1 Introduction

Obstetric violence (OV) is defined as abuse perpetuated by healthcare workers toward expectant women in the course of the birthing process, mainly in the form of disrespect, dehumanisation, medication abuse and other forms of bodily harm (1). OV contribute to maternal mortality by influencing women's choice not to seek facility-based birth, even when they might have risk factors (2–4). In this essay, we will review the evidence on OV in Ghana and how introducing Midwife-led care (MLC) could offer cost-effective solutions to OV in Ghana.

## 2 Approach

This article adopts a perspective to critically explore OV in Ghana and MLC as an approach to promote respectful and equitable maternity care. It does not generate primary data; instead, it synthesises and interprets existing evidence to inform policy and practice. Evidence was drawn from peer-reviewed qualitative and quantitative studies, systematic reviews, and policy documents from authoritative bodies such as the World Health Organisation (WHO) and the International Confederation of Midwives (ICM). Themes were organised and reported under clearly defined subheadings, including forms of obstetric violence, systemic and relational drivers, consequences for women's wellbeing and care-seeking, and mechanisms through which MLC may mitigate mistreatment during childbirth, and the effectiveness of MLC models, mainly in low- and middle-income countries and the Ghanaian context. The analysis focused on how continuity of care, women's autonomy, respectful communication, and culturally responsive practice—core features of MLC—may help reduce OV. Global evidence was interpreted alongside context-specific data from Ghana to enhance relevance for national health systems and maternal health policy.

### 2.1 Background

Maternity care encompasses the provision of care to women before, during, and after childbirth (5). As part of achieving Sustainable Development Goal (SDG) three, the role of the midwife cannot be understated. A report by the World Health Organisation (WHO) indicates that globally, a woman dies every two minutes through childbirth, with 287,000 maternal deaths recorded worldwide in 2020 (6). Most of these fatalities take place in developing countries. Furthermore, the report indicates that 70% of all maternal deaths were reported in Sub-Saharan Africa (6). This is primarily a result of inadequate maternity care planning, an unskilled workforce, and insufficient resources (7), which contribute to OV. Dealing with OV demands coordinated efforts through the MLC (8, 9), as the MLC is a global standard for improving maternity care. In addition, women are notably less likely to experience OV if their deliveries are cared for by a midwife instead of a nurse or community health nurse (10). This article is a perspective drawing on selected literature and contextual data from Ghana, rather than a primary community case study.

#### 2.1.1 Form of OV

OV exists in the form of lack of privacy, physical abuse, stigma and discrimination, non-existence of culturally sensitive treatment, verbal abuse and unwarranted detention in health facilities (10–14). Moreover, the widespread mistreatment of women during childbirth was highlighted globally, including physical abuse, non-consensual care, and discrimination by healthcare providers (15). In contrast, Bohren et al. (4) found that 41.6% of women reported experiencing physical or verbal abuse, stigma, or discrimination, offering specific prevalence data that reinforces the former studies' broader findings. Notably, younger, poorer, unemployed women with limited literacy skills, and unmarried women were identified as the most vulnerable to such mistreatment (4).

#### 2.1.2 Prevalence and causes of OV in Ghana

The concept of OV is an emerging concept in the global health perspective (10). The prevalence rate of OV in Ghana is estimated at 65–83% (10, 61). Further, an August 23rd report by the Ghana News Agency (GNA) indicates that 2/3 of all women have been exposed to OV during childbirth, mainly in the form of mistreatment and abuse (16). However, the causes of OV are multifaceted. These factors may include limited resources, heavy workloads, adolescent motherhood, midwives' status in the health system, power imbalances, type of facility, and low family income, particularly among women unable to afford bribes or medical costs (10, 14, 17–19). Yalley et al. (10) in their study, they noted that the risk of OV by nurses increases because they were not trained to provide maternity care. In addition, Bradley et al. (17) found that poor infrastructure, lack of material resources and shortage of staff compromised midwives' ability to provide the requisite care for women. Moyer et al. (20) reported a lack of adherence to traditional practices of family members and husbands of women present during delivery. Moreover, Yalley (21) found blame of midwives, fear, frustration, and termination of professional license in case of maternal death as causes of OV.

#### 2.1.3 Effect of OV on women

The effect of OV has always had negative consequences and usually has a lasting effect on the woman (22). A study by Vedam et al. (23) reported that socially, women who received midwifery care noted a rise in their sense of control and independence when it came to making decisions. Clinically, the effect of OV is huge on the mother and child. The fear of being humiliated at the health facility may result in home delivery and a lower likelihood of returning to the hospital for continuity of care (20). In addition, OV results in mistrust in healthcare, physical injuries to the mother and newborn, psychological trauma for the mother, foetal distress, raised caesarean delivery, tear in the vagina, excessive bleeding, and significant dangers posing a threat to the lives of mothers (24, 25). Similarly, Raj et al. (26) reported that women exposed to OV during childbirth are vulnerable to complications such as prolonged labour, obstructed labour, and postpartum haemorrhage. These complications put the lives of the women at risk.

OV has ramifications for the psychological health of women. Silveira et al. (24) found that postpartum depression was 1.6 times more likely in women who encountered disrespect and abuse during childbirth, particularly verbal abuse. This association was observed even in women without antenatal depressive symptoms, highlighting the impact of mistreatment during the childbirth experience (24). Other studies have reported that OV during childbirth leads to mental health consequences for women (27, 28), such as postpartum depression (29). Another effect of OV is utilisation-related, where women prefer home delivery under the care of unskilled birth attendants. Rude behaviour, poor treatment and disregard by health carers during antenatal care and labour discourage pregnant women from birthing at the health institution, and as a result, facilitate birthing at home (30). Relatedly, Garcia (31) concluded that obstetric violence leads to a loss of autonomy on matters of sexuality among women. Moreover, Kane et al. (32) contended that the fact that women are afraid of going to hospital facilities due to disrespect and abuse further leads to maternal mortality. To address this issue and decrease maternal mortality, it is essential to recognise and overcome obstacles that hinder access and compromise the quality of maternity care within the healthcare organisation. Therefore, increasing MLC is crucial to reducing OV.

#### 2.1.4 Role of midwifery-led care in reducing OV

The unique role of the midwife in reducing maternal deaths cannot be discounted, because they are instrumental in ensuring the safety of the mother and the baby (33). As a way of dealing with obstetric violence, an MLC has been recommended (34, 35). The International Confederation of Midwives (36) defines MLC as the midwife serving as the primary healthcare professional, tasked with planning, organising, and providing care to a woman from the early booking of antenatal visits to postnatal care. This also involves the provision of maternal care by qualified midwives (5), and utilising the same midwife throughout pregnancy and the postnatal period to ensure continuity of care (37). MLC has been demonstrated to provide respectful and culturally sensitive care and increase family involvement, women's trust, respect for privacy, effective communication and decrease hospitalisation (38–41). Furthermore, in comparative studies on freestanding midwifery units (FMU) and obstetric units (OU), women who chose FMU reported receiving high-quality care (42, 43). Based on these experiences, it is anticipated that OV will be less/reduced with MLC. Hence, addressing OV by implementing MLC will increase facility-based birthing, which will indirectly reduce maternal deaths in Ghana.

## 3 Contextual analysis

The maternal mortality rate in Ghana is estimated at 234 deaths per 100,000 births (44), however, per the WHO country-specific target, Ghana needs to meet a target of less than 140 deaths per 100,000 (45–47) births to achieve Agenda 2030 (48). This is among the highest in LMICs according to the Ghana Health Service (GHS) (49). Ghana has invested in various strategies to reduce maternal mortality, including life-saving skills training, Community-based Health Planning and Services (CHPS), free maternity care, targeted antenatal services, the national health insurance scheme, etc. (50, 51). However, despite these efforts, the country did not meet Millennium Development Goal 5. The proportion of skilled birth deliveries in Ghana ranges from 54% to 63%, contrasting with the higher percentage of women (97%) who avail themselves of antenatal care services (10). This discrepancy suggests a significant number of Ghanaian women opt for home births under the care of unskilled birth attendants, potentially contributing to the increased rate of maternal deaths in the country.

Maya et al. (11) investigated the prevalence of OV and reported that the primary forms of mistreatment identified encompass verbal and physical abuse, as well as abandonment and insufficient support during the second stage of labour, particularly among adolescent mothers. Furthermore, slapping and pinching were considered acceptable methods to “correct” disobedient behaviour and encourage pushing (11). Consequently, many women expressed a reluctance to choose health facilities for future childbirth experiences, either based on their encounters with mistreatment or upon learning about mistreatment experiences from other women (11). Again, 65.1% of HIV-negative women experienced OV, slightly higher than 61% observed in HIV-positive women (52). These findings indicate that OV is a major concern in Ghana, irrespective of the status of the woman. In the Northern Region of Ghana, Moyer and her colleagues reported physical abuse (hitting women in labour, slapping, and kicking), verbal abuse (shouting, insulting, and speaking harshly, use of inappropriate words), neglect, discrimination based on the poverty level of the women, non-conformity to traditional customs during surrounding childbirth (preventing women from assuming a squatting position during labour and prohibiting women from retaining the placenta after childbirth) as the most common form of OV (20).

Dzomeku et al. (53) revealed that OV was pervasive in their qualitative study in two regions from the perspective of midwives. The study found that delivering insufficient care, neglecting patient-centred approaches, verbal maltreatment (insulting and shouting at childbearing women), bodily harm (hitting, whipping, and striking of pregnant women), and psychological mistreatment (disregarding, abandoning, delivering care that lacks a person-centred approach) were common. Again, from the perspective of midwifery students, Rominski et al. (54) reported justification of OV to include overwork, lack of resources, the culture of blaming midwives for maternal mortality, and disrespect towards midwives as causes of obstetric violence. Similarly, lack of skilled midwives, inadequate resources, competence of healthcare workers, and hygienic conditions at the hospital were the findings of a study in the Volta region (13). In Ghana, inadequate wage coerces some midwives to engage in trading and informal extortion, such as selling diapers, water, or clothing, which can result in neglect, discrimination, partiality, and even the detention of women unable to pay for items (55). A midwife-to-patient ratio of 1:560 in Ghana aggravates staff shortages and heavy workloads, contributing to the mistreatment of women (55). Additionally, the health system's hierarchical structure positions doctors and inflexible protocols as the definitive authority, subordinating midwives despite their specialised training. This marginalisation often motivates midwives to assert power over women, occasionally through bullying and offensive practices (56). Lately, Yalley (21) recounted that OV was common within health care and a routine. Midwives viewed OV as a delivery strategy and a form of help to the client. Shouting, forced medical care, stigmatisation, non-consented care, denial of birth companions, and denial of preferred birth Position were not uncommon (21). However, a study in Ghana on midwife-led obstetric triage training concluded that it enhances knowledge and practice, tackles the third delay and decreases avertible pregnancy-related deaths (57). Again, from LMICs' perspectives, MLC decreased both maternal and neonatal death (58). Moreover, managing OV among mothers is important to reduce the effects on women. This can result in better care, good interpersonal relationships, and positive outcomes of pregnancy (19). Therefore, replicating similar evidence in Ghana will help combat rampant OV in maternity wards nationwide.

## 4 Implications for future applications

### 4.1 Policy level

⮚ Policy must be targeted at resourcing health facilities by providing the resources needed for work, and by increasing the midwives-patient ratio to deal with the stress experienced by the midwives.
⮚ Stakeholders engagement and awareness, especially among obstetricians, are needed to reduce resistance from them.
⮚ The GHS and MoH should incorporate traditional practices and cultural beliefs that surround childbirth to enhance respect for women.

### 4.2 System level

⮚ Introduce midwife-led care in primary settings, which implements the bio-psycho-social philosophy of care in an integrated service where transfer to secondary or tertiary care is well organised.
⮚ Enact laws and regulations mandating midwives to be responsible for providing maternal services to pregnant women with uncomplicated pregnancies.
⮚ Implement and adopt a modified World Health Organisation Safe Childbirth Checklist (59) such as using a mobile app and posters to include Respectful Maternity Care.

### 4.3 Provider level

⮚ Introduce training in all settings to understand the skills in personalised care as an antidote to dehumanised care resulting from the medical industrial philosophy of care.
⮚ Scale training of midwives to provide service at the primary level, especially in health centres and Community-based Health Planning and Services (CHPS) compounds.
⮚ Provide ongoing in-service interactive skills workshops using different methods like presentations, role-playing, demonstrations, case studies, individual readings, videos, and hospital visits for midwives to stay updated with the latest maternal care practices and technologies (59).

## 5 Conceptual constraints

Opposition from obstetricians is likely to increase if midwives are given autonomy to manage uncomplicated pregnancies. Therefore, it is essential to engage stakeholders and present evidence-based outcomes demonstrating the benefits of midwife-led care. Additionally, subsidising fees for continuing professional development and providing e-learning platforms will enable midwives in remote and underserved areas to enhance their skills. Moreover, the Ghanaian government should provide sufficient funds to help establish midwifery units. Furthermore, non-cooperation among disciplines could contribute to OV. Yet, collaboration strengthens interpersonal relationships, fosters trust, and provides access to varied competencies within an organisation (60). Adopting a collaborative care model can therefore reduce strict hierarchies, promote reciprocal respect among healthcare providers, and enhance teamwork and collective accountability (56).

## 6 Conclusion

Although OV and maltreatment of women during childbirth are universal phenomena, the prevalence in Ghana is high. However, Midwife-led care (MLC) is valid and evidence-based, proven effective in multiple LMICs. It directly targets determinants of obstetric violence—disrespect, poor communication, and lack of autonomy. In Ghana, implementation is feasible but requires system readiness and stakeholder engagement.

## Data Availability

The original contributions presented in the study are included in the article/Supplementary Material, further inquiries can be directed to the corresponding authors.
